# Mass spectrometry in epidemiological studies: What are the key considerations?

**DOI:** 10.1007/s10654-016-0195-x

**Published:** 2016-08-26

**Authors:** Abbas Dehghan

**Affiliations:** 1 Department of Epidemiology and Biostatistics, School of Public Health, Imperial College London, London, UK; 2 Department of Epidemiology, Erasmus University Medical Center, P.O. Box 2040, 3000 CA Rotterdam, The Netherlands

The incursion of “-omics” (e.g. genomics, epi-genomics, transcriptomics and metabolomics) in epidemiological studies has revolutionized the practice of epidemiology. Comprehensive and accurate assessment of environmental factors and health status has been a perpetual challenge for epidemiology. Omics are expected to broaden the domain of exposure assessment and allow more detailed evaluation of health status, providing further details and additional platforms of understanding. In recent years, novel -omics approaches have enabled epidemiologists to quantify characteristics that were not previously within their reach and to investigate features that up to a few years ago were not even in their sight. Moreover, the agnostic approach that is used in -omics has enabled epidemiology to move beyond current biological knowledge and contribute to build upon it. This has provided an unprecedented opportunity for epidemiology to contribute to cutting-edge and groundbreaking science by exploring pathways and mechanisms in more details and advance our understanding of health determinants at molecular level.

Despite the spectacular promises of -omics that have put epidemiology in the limelight, addressing the concomitant criticisms continues to be challenging. Implementing -omics on a large scale is an expensive effort and thus the investments are substantial. Stakeholders are eager to see the immediate clinical or public health applications of the investments. Nevertheless, current findings are too basic to be immediately used in practice and efforts for clinical applications are still in initial phases. Genome-wide association studies (GWAS) were one of the first –omics technologies that quickly became popular in epidemiological studies and turned out to be fruitful in locating potential genes for complex traits and disorders. Nevertheless, the discoveries have hardly led to a clinically used drug. Personalized medicine and its successor, precision medicine, were thought to be fields where GWAS could contribute in the short term, for example by risk stratification or individualized treatment based on genetic information. However, efforts to use GWAS findings in personalized medicine have not yet been successful due to the small contribution of the identified genetic variants to the risk of diseases. Metabolomics, another -omics that studies the small molecules, however, appears to be promising and could potentially tackle these challenges. Metabolites contain more information on the health status compared to other -omics due to their closer proximity to the phenotype. Moreover, in comparison to genetic information that is constant throughout the life course, the metabolome has the advantage of a dynamic nature and including real-time information on the health status.

Metabolomics is either targeted or untargeted. Targeted metabolomics aims to measure a defined group of chemically known and biochemically annotated metabolites. Untargeted metabolomics, however, is a comprehensive analysis of all quantifiable molecules in a sample including unknowns chemical. Although targeted metabolomics has already been used in a number of epidemiological studies, applying untargeted metabolomics to large-scale epidemiological studies is a relatively novel application for this technique. Its use, though, is rising due to a combination of improved laboratory techniques, innovative statistical methods, and an associated reduction in costs.

Two of the most commonly used technologies for metabolomics are nuclear magnetic resonance spectroscopy (NMR) and mass spectrometry (MS). NMR relies on the fact that certain nuclei (^1^H, ^13^C, or ^31^P) possess the property of magnetic spin and when placed inside a magnetic field can adopt different energy levels that can be observed using radiofrequency waves. MS, however, measures the mass-to-charge ratio of ions formed from molecules, usually separated by chromatography. ^1^H-NMR spectroscopy has been widely used in previous studies due to its reproducibility, fast analysis, continuous performance, and feasibility to study all organic molecules since they all have protons. However, ^1^H-NMR cannot measure metabolites that are present in low concentrations. Such low concentration metabolites can only be identified and quantified using more sensitive mass spectrometry (MS) methods. Despite the fact that NMR has so far been the dominant technology used in epidemiological studies, MS may be the main method that will be implemented in epidemiological studies in the future.

In recent years the European Journal of Epidemiology (EJE) has paid special attention to the flourishing field of “-omics epidemiology” by publishing papers in this domain covering topics such as molecular pathology [1], gene-environment interaction [2], population admixture in association studies [3], and metabolomics [4]. In this issue of the EJE Lind and colleagues present a comprehensive yet uncomplicated and clear overview of MS methods and their applications in epidemiological studies. The paper describes technical details of the method, reviews a number of applications with a focus on epidemiological studies, goes over key sampling and analytical considerations in large scale studies, reviews the relevant statistical methods and highlights major advances in the field.

Many epidemiological studies may currently not see metabolomics and especially MS as a pressing need. Nevertheless, the wide range of applications for MS, ranging from exposure measurements in environmental epidemiology to risk stratification and etiological research in classical epidemiology will make the technology essential in long run. It should be noted that the samples that will be assayed in future are the ones that are collected today. The validity of the future quantifications, therefore, will depend on proper collection, processing and storage of these samples now. In other words, epidemiologic studies ought to keep their protocols up-to-date to enhance their future application in upcoming technologies. In this regard Lind and colleagues offer a comprehensive description of sampling for metabolomics studies that could be of interest to a wide range of epidemiologists currently collecting population resources even if there is no plan to implement omics in their studies in the near future.

Implementing metabolomics techniques such as MS through agnostic approaches calls for collaborative projects aiming either to extend the discovery by combining several studies or to seek validity through replication in independent samples. Unlike genomics and epigenomics, technical variability is larger in MS data. Moreover, the assays, quality control set ups, data preprocessing and metabolite identification/quantification are not well standardized and homogenized. The variability produced by these diversities will lead to major challenges in combining and comparing the data or translating them from one platform to another when the assays are done in different centers or in several batches within the same lab. Papers such as the one by Lind and colleagues that catalogue a standard practice could be an initial step towards an urgently needed standardization of the methods and procedures.

MS is not a novel technique but applying it to epidemiological studies is a novel advent. The techniques are evolving and the field is emerging. It is likely that the technical challenges that have so far hampered the extensive application of metabolomics and MS techniques at the population level will be reduced or tackled in the coming years. Epidemiological resources will provide the infrastructure for such a revolution in population-based research and such a revolution will let the investments that have made in epidemiological studies flourish once again.
